# Recent Advances in the Mechanism Research and Clinical Treatment of Anti-Angiogenesis in Biliary Tract Cancer

**DOI:** 10.3389/fonc.2021.777617

**Published:** 2021-10-28

**Authors:** Yue Wang, Tianli Chen, Kangshuai Li, Wentao Mu, Zengli Liu, Anda Shi, Jialiang Liu, Wei Zhao, Shuo Lian, Shaohui Huang, Chang Pan, Zongli Zhang

**Affiliations:** ^1^ Department of General Surgery, Qilu Hospital, Cheeloo College of Medicine, Shandong University, Jinan, China; ^2^ Department of Emergency, Qilu Hospital, Cheeloo College of Medicine, Shandong University, Jinan, China

**Keywords:** angiogenesis, gallbladder carcinoma, biliary tract cancers, cholangiocarcinoma, targeted therapy, mechanism, antiangiogenic therapy

## Abstract

Biliary tract cancers (BTCs), including cholangiocarcinoma (CCA) and gallbladder cancer (GC), are malignancies originating from the biliary tract with poor prognosis. In the early stage of BTCs, surgery is the only choice for cure. Unfortunately, most patients with BTC are diagnosed at an advanced stage and lose the opportunity for surgery. For many advanced solid tumors, antiangiogenic therapy has achieved encouraging results. While most clinical studies on antiangiogenic therapy in advanced BTCs have shown an excellent disease control rate (DCR), the improvement in overall survival (OS) is controversial. Understanding how the relevant signaling molecules influence the angiogenic response and the functional interaction is necessary for the formulation of new treatment regimens and the selection of enrolled patients. In this review, we aim to summarize and discuss the latest advances in antiangeogenesis for BTCs, mainly focusing on the molecular mechanism of angiogenesis in BTCs and the therapeutic effects from clinical trials. Furthermore, the horizon of antiangiogenesis for BTCs is highlighted.

## Introduction

Biliary tract cancers (BTCs) are a diverse group of malignancies originating in the biliary epithelium (1). According to their anatomical site of origin, BTCs are divided into cholangiocarcinoma (CCA) and gallbladder cancer (GC). CCAs are further classified as intrahepatic CCA (iCCA), perihilar CCA (pCCA), and distal CCA (dCCA) (2). PCCA and dCAA are also collectively called eCCA. BTCs account for only 3% of all gastrointestinal cancers, and the incidence of BTCs has increased over the past few decades (3). At present, surgical resection is still the only radical cure for BTCs (4). However, because the symptoms of BTCs in the early stage are atypical, most cases are diagnosed at an advanced stage of disease and therefore lose the opportunity for radical surgical treatment. For patients with advanced unresectable or metastatic BTCs, systemic therapy might be the only beneficial treatment option (5). Unfortunately, due to the insensitivity to systemic therapies such as chemotherapy, the outcome of advanced and metastatic BTCs is unsatisfactory, with a 5-year survival rate of approximately 10%. Therefore, new therapies for BTC are urgently needed to improve the OS rate (6). In the era of precise treatment, antiangiogenic drugs are a main component of targeted therapy. However, the application of antiangiogenic therapy in BTC lacks consensus, and the criteria to select appropriate patients for antiangiogenic therapy have not been studied (7).

Metastasis is the main cause of individual death during tumor progression. There is sufficient evidence that tumor neovascularization is the pathological basis and necessary condition for the growth and metastasis of solid tumors (8). Tumor ischemia, on the one hand, affects the nutritional supply of the tumor, but on the other hand, it also impedes drug accessibility to the tumor and even promotes the selection of more aggressive tumor cells. Some scholars believe that promoting the normalization of tumor blood vessels will be an effective treatment (7).

Tumor-associated angiogenesis is active, and microvascular density is increased in BTCs, which contributes to the low cure and high recurrence rates after surgical resection. Microvessel density (MVD) is an indicator of tumor-driven neovascularization. MVD is significantly associated with survival and prognosis in GC, iCCA and extrahepatic cholangiocarcinoma (eCCA) (9–11). Studies confirmed that higher MVD was associated with advanced tumor stage and lower tumor resection rate and that MVD was an independent prognostic factor in a multivariate analysis. The 5-year survival rate of the high MVD group (2.2%) was significantly lower than that of the low MVD group (42.1%) (11), which suggested that the prognosis of BTCs is closely related to tumor angiogenesis. Although some antiangiogenic drugs have been approved for clinical trials in BTCs, the results are unsatisfactory. At present, the mechanism of tumor angiogenesis in BTCs is not clear, and the relevant targeted therapy needs to be further studied (12). This review summarizes the current consensus on tumor angiogenesis, with a focus on angiogenesis as the driving force in BTC development, and the status of the research and application of antiangiogenic therapy in BTCs.

## Tumor Angiogenesis and Tumor Vascular Normalization

Tumor blood vessels are characterized by structural disorder, incomplete wall structure and high permeability, which can lead to local hypoperfusion of the tumor (13). Thus, it is difficult for either oxygen or drugs transported through blood vessels to enter the tumor parenchyma, which will greatly reduce the effectiveness of radiotherapy, chemotherapy and immunotherapy (14–16). The ionizing radiation of radiotherapy can locally produce reactive oxygen species (ROS) in the presence of oxygen. ROS can damage DNA and directly result in the death of tumor cells (17). However, the low-oxygen tumor microenvironment weakens the effect of this treatment. Systemic chemotherapy drugs need to attach to the local tumor area through the blood circulation system. A low perfusion state and increased interstitial pressure prevent drugs from attaching to the tumor area or reduce the amount of drugs entering the tumor parenchyma, thereby affecting the efficacy of chemotherapy. For the tumor itself, the lack of effective blood perfusion leads to a hypoxic tumor microenvironment (18). Hypoxia can activates the HIF (Hypoxia-inducible factor) signaling pathway, which promotes tumor cells to overexpress VEGF to induce tumor angiogenesis (19); In addition, tumor cells with more aggressive and metastatic ability will be screened out because of the harsh tumor microenvironment (20). The RhoA-ROCK1 signaling can be activated by HIF to enhance cell motility (21). Hypoxia increases hypermethylation of tumor suppressor gene, which makes epigenetic aberration and then promotes tumor growth and metastasis (22). Furthermore, hypoxia inhibits tumor immunity by inhibiting cytotoxic T cell activity, promoting local tumor recruitment of Treg cells, and inhibiting the synthesis of pro-inflammatory cytokines. These effects involve a series of factors including cAMP, HIF, COX2, SDF1, IL-10, etc. (23–26).

In the past, antiangiogenic therapy was thought to work by blocking the pathway of tumor angiogenesis and cutting off the nutrient supply to the tumor. However, with clinical research in recent years, it has been found that the effect of antiangiogenic therapy is limited, and some patients are more prone to tumor metastasis after antiangiogenic therapy (27). This is because antiangiogenic drugs severely degenerate tumor blood vessels, while blocking the nutritional supply of tumor, the supply of drugs and oxygen also been hindered. Such conditions can help tumor tissue resist the effects of radiotherapy and chemotherapy, and further deepen the hypoxia condition of tumor microenvironment (28). As mentioned above, hypoxia increases tumor malignant phenotype and inhibits tumor immunity, which is obviously not conducive to tumor therapy. Until the theory of tumor vascular normalization was proposed in 2005 (13), the purpose of antiangiogenic therapy has not changed from degrading tumor blood vessels to promoting the maturation of tumor blood vessels, thereby improving local blood perfusion and material transportation in the tumor microenvironment. This notion partly addresses the limitations of antiangiogenic therapy and provides a theoretical basis for antiangiogenic therapy combined with chemotherapy or targeted therapy. After antiangiogenic treatment, there will be a specific time window. At this time, the tumor blood vessels will be temporarily and reversibly normalized, and drugs are easier to enter the tumor microenvironment (29).

Inhibition of tumor angiogenesis and induction of tumor vascular normalization are of great significance in the treatment of tumors (30). Unfortunately, the existing antiangiogenic therapies have not shown promising therapeutic effects, especially in the field of BTCs. Existing studies have shown that anti-VEGF therapy not only inhibits tumor angiogenesis, but also promotes normalization of tumor blood vessels (31). There are also reports showing that treatments targeting VEGF can increase the invasion and metastatic phenotype of tumors (32). These contradictory results are believed to be related to the complex regulatory network of tumor angiogenesis. Vascular endothelial protein tyrosine phosphatase (VE-PTP) inhibitors can mature tumor vessels by activating Tie-2 (33). Overexpression of proteins such as R-RAS and HRG which contribute to vascular maturation can normalize abnormal tumor vessels (34, 35). These findings provide new targets for tumor vascular normalization. In addition to the drug itself, the dose and duration of drugs are crucial for abnormal tumor vascular development to different outcomes, tumor vascular normalization and tumor vascular degeneration. While methods to assess the time window of tumor vessel normalization are still scarce, and the control of drug dose and time is not ideal (36). Furthermore, the mechanism of angiogenesis is ambiguous in tumors, and there are no therapeutic targets that can bypass normal blood vessels. Therefore, further research is needed to clarify the mechanism and explore more effective targets and treatment strategies (7).

## Factors and Mechanisms That Regulate Angiogenesis in BTCs

**Figure 1:**
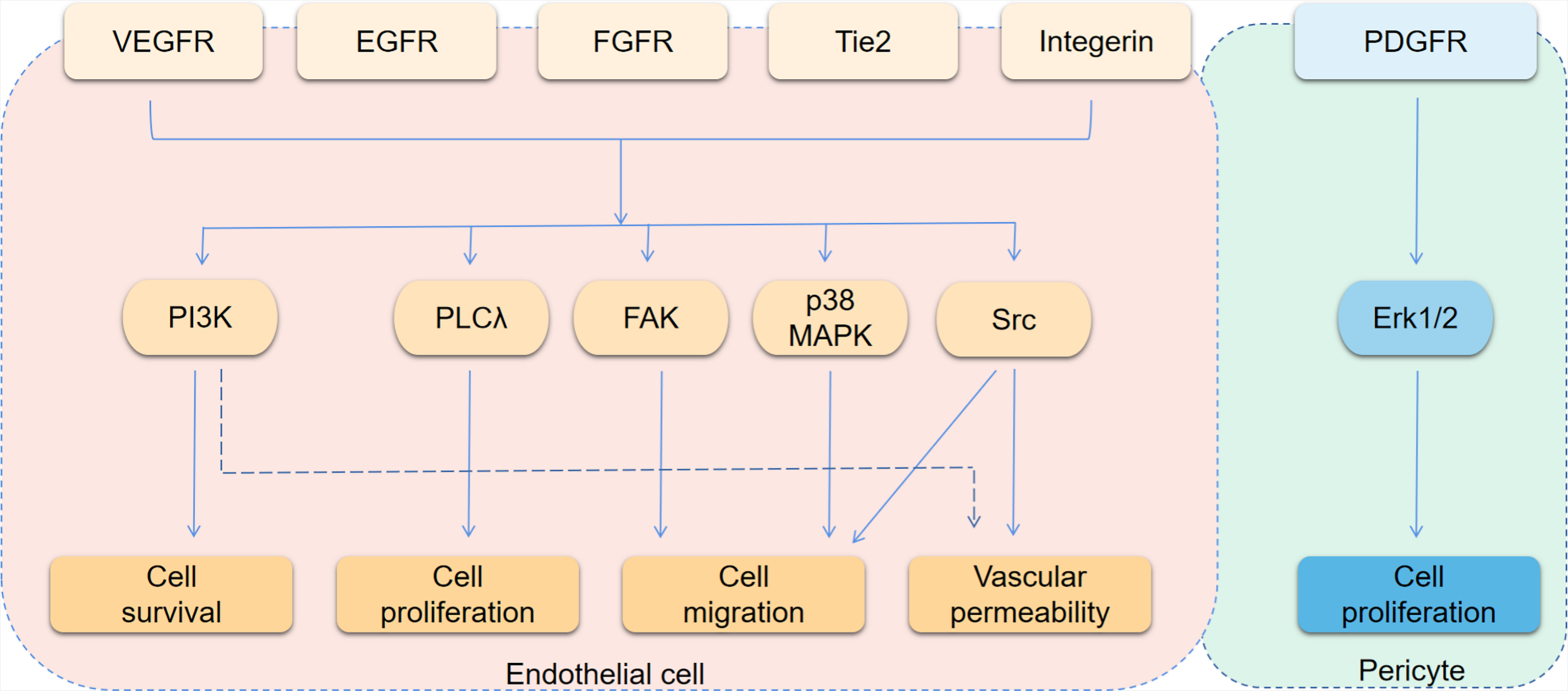
Angiogenesis signaling pathways. The figure shows the main downstream pathways of receptors associated with angiogenesis. The main phenotypes affected by each pathway are indicated by arrows.

The growth of solid tumors is accompanied by tumor angiogenesis, and it is reasonable that anti-angiogenic therapy should be an important part of anti-tumor therapy. However, therapeutic regimens targeting VEGF/VEGFR have consistently failed to provide encouraging results (37). The main reasons is that there are many ways to promote angiogenesis (**Figure 1**). VEGF-dependent or VEGF-independent neovascularization and angiogenesis mimicry can all provide blood vessels for tumor tissues. Inhibition of one pathway leads to compensatory activation of other pathways. Besides, classical proangiogenic pathways, such as the VEGF/VEGFR pathway, also play a role in normal tissue. That makes treatment easier to be interrupted by on-target off-tumor toxicities (38). Therefore, it is critical to find tumor-specific antiangiogenic targets and the common pathways in different angiogenesis mechanisms. Understanding the pro- and anti-angiogenesis factors as well as their interaction and molecular mechanisms is essential for the development of durable and effective anti-angiogenesis drugs (39). In the following content, we will describe the role of pro-angiogenic and anti-angiogenic factors in BTCs, especially their regulatory mechanism.

## VEGF/VEGFR Signaling Pathway

VEGF is a growth factor with the strongest angiogenic activity. The VEGF growth factor family includes VEGF-A, -B, -C, -D, -E, -F and placental growth factor (40). Among these types of VEGF, VEGF-A is generally believed to play the most obvious role in promoting angiogenesis. During mouse embryonic development, VEGF or VEGF receptor (VEGFR) gene deletion leads to embryonic death due to angiogenesis disorder. Additionally, VEGF plays an important role in tumor angiogenesis. VEGF is overexpressed in many epithelial tumor cells. A large number of studies have reported that the level of VEGF in peripheral blood is directly related to tumor prognosis (41).

VEGF mainly acts through the corresponding receptors Flt-1 and KDR, also known as VEGFR-1 and VEGFR-2 (42). Both Flt-1 and KDR are receptors for tyrosine kinase receptors (RTKs). The binding of VEGF and KDR activates the MAPK signaling pathway and promotes endothelial cell proliferation and angiogenesis. The function of Flt-1 is more complicated. In addition to promoting angiogenesis, the combination of VEGF can also activate matrix metalloproteinases (MMPs). The increased expression of VEGF in tumor tissues is related to hypoxia-inducible factors, which can promote the expression of VEGF. The traditional concept states that VEGFR is specifically expressed in endothelial cells. However, an increasing number of recent studies have shown that tumor cells can also express VEGFR. VEGF secreted by tumor cells can promote angiogenesis and tumor cell proliferation through paracrine (acting on endothelial cells VEGFR) and autocrine (acting on the VEGFR of tumor cells) pathways.

**Figure 2:**
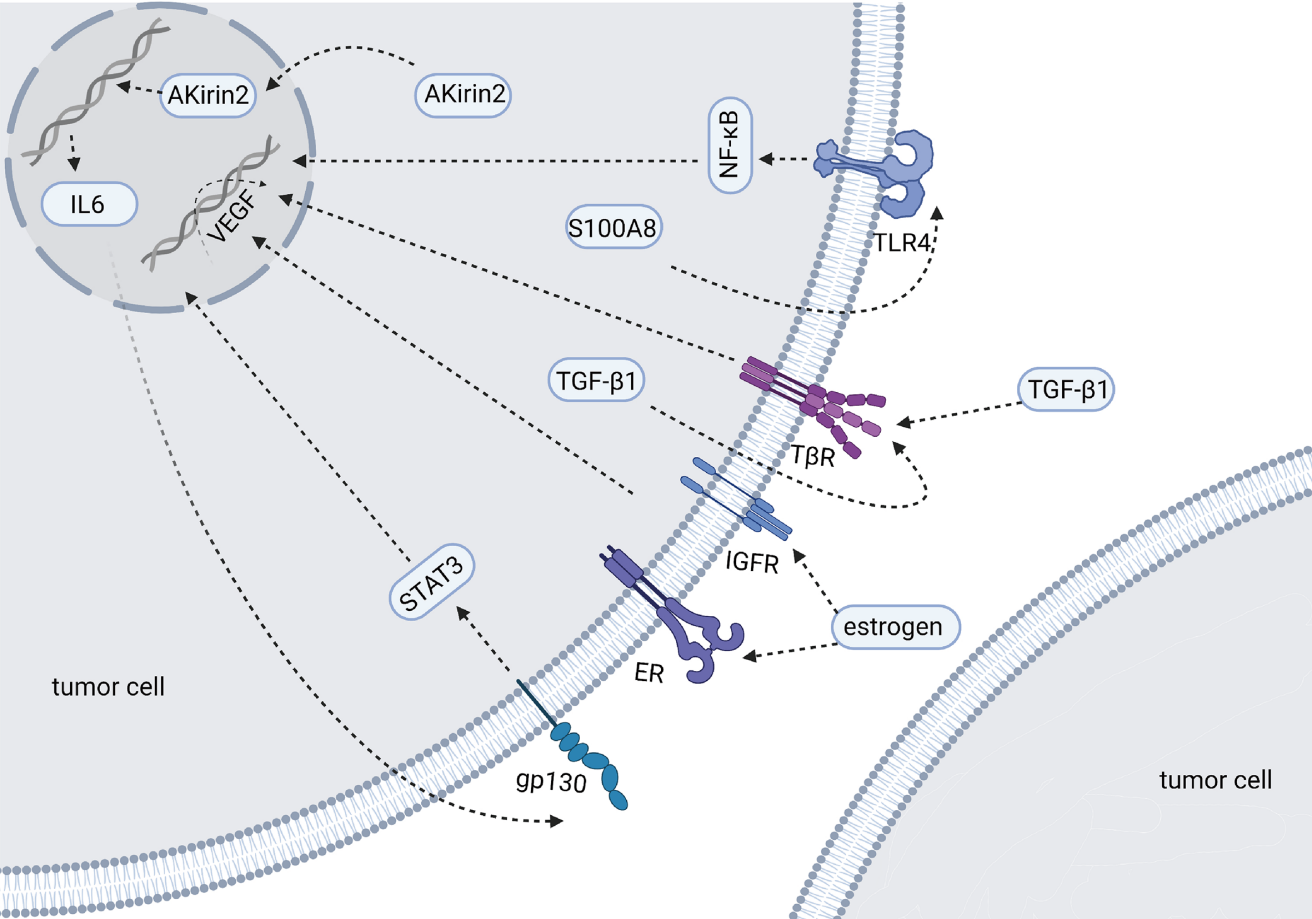
Molecules and mechanisms regulating the expression of VEGF in BTCs (Created in BioRender.com). The figure mainly shows the factors have been reported to affect the expression of VEGF in BTCs. These factors are overexpressed in tumor cells and their downstream signaling pathways of affecting VEGF have been described by researchers.

Some researchers examined VEGF expression in four CCA cell lines and their culture supernatants. The results show that CCA cells can express and secrete VEGF (43). VEGF can be detected in bile and can be used as a diagnostic and predictive biomarker for different biliary diseases (44). Studies have shown that the positive expression rates of VEGF in clinical samples of iCCA, eCCA and GC are 53.8%, 59.2% and 56.3% (45–47). The overexpression of VEGF is related to the intrahepatic metastasis of iCCA (P=0.0224), while there was no significant correlation between VEGF and the clinical features of eCCA in this study (46). Other studies have shown that the expression of VEGF-A tends to increase in hypervascularized eCCA, but it did not reach statistical significance (P=0.08) (48). Further studies on eCCA have shown that, compared with pCCA, the positive expression rate of VEGF-A in dCCA is higher (69% *vs.* 25%, P <0.0001), and it is related to an increase in microvessel density (49). When VEGF was neutralized by adding 10 mg/mL anti-VEGF antibody to the medium, vascular endothelial cells decreased to 63.8% of the control group (P <0.02) (43). Any factor that destroys the expression of VEGF or VEGFR may affect the angiogenesis of BTCs. The following factors, hormones or drugs that may affect the VEGF signaling pathway were identified in the study of BTCs (**Figure 2**). TGF-β1 is expressed in tumor cells or surrounding mesenchymal cells, and immunostaining shows that its receptors TβR-I and TβR-II are strongly positively expressed in tumor cells. Studies have shown that TGFβ1 can promote the expression of VEGF in tumor cells through autocrine or paracrine modes and then affect tumor angiogenesis (50). S100A8 is highly expressed in CCA cells and increases the secretion of VEGF by activating the Toll-like receptor 4 (TLR4)/NF-κB pathway, thereby inducing the migration of vascular endothelial cells (51). Studies also show that COX-2 is related to angiogenesis. COX-2 is highly expressed in CCA tissues, especially advanced CCA (52). COX-2 inhibitors have been approved for adjuvant treatment of CCA, but *in vitro* experiments show that they have no inhibitory effect on the growth of tumor cells. COX-2 inhibitors can inhibit the expression and secretion of VEGF-C, thereby affecting the invasion of cholangiocarcinoma (52). MiR-101 can also inhibit COX-2 or directly target the 3’ untranslated region of VEGF mRNA to inhibit VEGF transcription (53). AKirin2 is overexpressed in CCA and promotes VEGF-A expression by activating the IL-6/STAT3 signaling pathway. This process can be inhibited by miR-490-3p (54). The highly conserved cell surface protein B7-H3 is reported to correlate with pathological rather than physiological angiogenesis and is regarded as an attractive target for the selective destruction of tumor vasculature (55). Estrogen can significantly increase the expression and secretion of VEGF-A in CCA cells. This effect is partially inhibited by estrogen receptor antagonists and completely blocked when used in combination with IGF1-R blocking antibodies (56). Tumor necrosis factor-α (TNF-α) can promote or inhibit endothelial cell growth and angiogenesis, depending on the cell condition. Treatment with lupeol or stigmasterol significantly reduced the secretion of TNF-α in umbilical vein endothelial cells, and then, the transcription level of VEGFR-2 decreased, which interfered with tumor angiogenesis by inhibiting VEGF signaling (57). HMGB1 induces angiogenesis by promoting the expression of VEGFR-2 in vascular endothelial cells (58). Histamine can increase the expression of VEGF-A/-C. Histamine stimulation has an effect on the angiogenesis observed in the tumor microenvironment. This effect can be inhibited by the HDC inhibitor α-methyl-DL-histidine dihydrochloride or the H1HR antagonist terfenadine (59). In addition, a drug, phenformin, can increase the expression and secretion of VEGF (60).

VEGF also has a close relationship with precancerous lesions. There are stem cells called hepatic stem/progenitor cells (HPCs) around the biliary tree. In primary biliary cirrhosis (PBC), a precancerous lesion of CCA, HPC is activated, and VEGF-A and VEGF-C are highly expressed (61). Higher angiogenesis can be observed in PBC samples. These studies indicate that the VEGF/VEGFR signaling pathway and angiogenesis may play an important role in the occurrence and development of BTCs.

## PDGF/PDGFR Signaling Pathway

The platelet-derived growth factor family (PDGF) regulates angiogenesis in tumors, and four family members have been identified, including PDGF-A, B, C, and D (62). PDGF can be synthesized and secreted by platelets, smooth muscle cells, vascular endothelial cells, pericytes and tumor cells. As a growth factor, PDGF combines with its receptor PDGFR to promote the growth of pericytes, vascular endothelial cells, fibroblasts, vascular smooth muscle cells and tumor cells by activating the Erk1/2 signalling pathway (63). PDGF promotes tumor cells migration by activating the p38/MAPK signalling pathway and overexpressing the MMPs. PDGF can also promote the directional migration and vascular envelopment of pericytes, thereby regulating the maturation and stability of tumor blood vessels. PDGF has been found to be highly expressed in a variety of solid tumors and as a predictor of poor prognosis. In biliary tumors, PDGF has been shown to be highly expressed and associated with poor prognosis (64, 65). Excessive activation of platelets has been found in CCA, which may be the source of PDGF in CCA (65). TCF-21 has been identified as a tumor suppressor gene in a variety of tumors, including CCA. When TCF-21 is overexpressed in CCA cells, it can inhibit the expression of PDGF (66).

## Ang-TIE-2 Signaling Pathway

Another important group of angiogenesis regulators is the angiopoietin family, including Ang1 and Ang2, which act through their receptor Tie-2. In recent years, this family of proteins has received increasing attention (67). When VEGF-mediated angiogenesis is blocked, the upregulation of Ang expression has been shown to be part of the angiogenesis rescue response, leading to accelerated tumor metastasis. Ang1 and Ang2 have opposite effects. Ang1 is the agonist ligand TiE-2 (68), which activates downstream pathways after phosphorylation of the receptor, protects blood vessels, maintains endothelial cell survival, and inhibits inflammation and vascular leakage. Ang1 can be expressed in a variety of cells, such as pericytes, smooth muscle cells and fibroblasts. Ang2, which is mainly expressed in vascular endothelial cells, has the opposite effect as Ang1. Ang2 is an inhibitory ligand of TiE-2 that can block Ang1-induced TiE-2 activation. Ang2 can also destroy vascular stability and promote inflammation and leakage (69). Therefore, both Ang1 and Ang2 play important roles in vascular remodeling and angiogenesis (70).

Ang1 has been shown to be highly expressed in a variety of tumor cells. Tumor-associated endothelial cells express high levels of Ang2, and the Ang2 concentration in peripheral blood is thought to be related to the tumor progression of BTCs (71). The TIE-2 receptor has also been detected in the tumor vascular endothelium (68). Although reported to promote tumor growth, Ang1 usually exerts an antitumor effect. In a study of biliary tract tumors, a team found that the expression of Ang1 in pCCA was negatively correlated with the metastasis rate, and the presence of TIE-2-expanded monocytes (Tems) in tumor tissues was associated with a lower recurrence rate (70). Ang2 expression was correlated with higher MVD in CCA (P=0.015). When both Ang2 and VEGF are positive, the MVD of CCA tissue is significantly increased (45).

## bFGF/FGFR Signaling Pathway

There are 22 members of the FGF family, from FGF-1 to FGF-23, except for FGF15 (human FGF19 and mouse FGF15 are homologous) (72). Among them, basic fibroblast growth factor (bFGF, also called FGF2) can affect vascular endothelial cells and stimulate angiogenesis. FGF receptor (FGFR) is expressed in endothelial cells. bFGF has been shown to be overexpressed in CCA (72, 73). Four CCA cell lines and their culture media were tested, and it was found that two of them can express and secrete bFGF, and all of them can express FGFR-1. The addition of anti-bFGF neutralizing antibody did not affect the proliferation of CCA cells but reduced the vascular endothelial cells to 58.9% of the control group (P<0.001). These results indicate that bFGF can affect the survival of the vascular endothelium in the form of paracrine signaling (43).

## Apelin/APLNR Signaling Pathway

The apelin/APLNR axis plays important roles in regulating blood pressure and cardiovascular disease and in regulating angiogenesis and the endothelial cell response to hypoxia (74, 75). Studies have shown that high expression of apelin can promote tumor angiogenesis in malignant tumors such as lung and liver cancers (76, 77). In CCA, researchers found that high expression levels of apelin and APLNR promote CCA cell proliferation and angiogenesis. Exogenous apelin stimulation can significantly increase the expression of angiogenesis factors (VEGF and Ang). Anti-APLNR reduces not only the expression of angiogenesis factors (VEGF and Ang) but also the expression of vimentin, MMP-9 and MMP-3 (78). These results suggest that the apelin/APLNR axis plays an important role in tumor angiogenesis. The mechanism regulating the expression of apelin/APLNR is not clear, but it has been extensively studied in different tumors. Apelin expression is related to oral squamous cell carcinoma hypoxia (79), and circulating apelin concentration is related to C-reactive protein in gastric and esophageal cancers (80). In prostate cancer, the level of apelin is regulated by microRNA-224 (81). Further research is needed in CCA.

## Other Factors and Mechanism

MMP can degrade the extracellular matrix and promote tumor angiogenesis. The expression of MMP2/MMP9 is increased in CCA and associated with poor prognosis. The overexpression of PDGF can contribute to the overexpression of MMP2/MMP9 (65). The roles of TSP-1 in tumor angiogenesis and tumor progression are still controversial. The expression of TSP-1 is associated with a significant decrease in MVD levels in CCA (45), which suggests that TSP-1 may play a role in inhibiting angiogenesis in CCA. However, the incidence of intrahepatic metastasis is higher when TSP-1 is positive (45). In a study of iCCA, TSP-1 was also positively correlated with lymphatic invasion (82). LOXL1 is a classic member of the LOX family. It is overexpressed in iCCA and can be secreted outside the cell. LOXL1 protein can bind to the exposed RGD domain of FBLN5, then the complex can bind to Integrin alpha V beta 3 on the surface of vascular endothelial cells and promotes angiogenesis *via* the downstream FAK and MAPK signaling pathways (83). In addition, angiostatin and endostatin can also inhibit angiogenesis (84, 85). Recombinant human endostatin (ENDOSTAR) can act on a variety of cell signaling pathways. It reduces tumor angiogenesis-related proteins and inhibits tumor lymphangiogenesis to inhibit angiogenesis. Moreover, it has been shown to have a good therapeutic effect on nonsmall cell lung cancer (46). Recent studies have shown that recombinant human endostatin can bring clinical benefits to patients with advanced cervical cancer. However, the expression and role of angiostatin in CCA remain unclear. Integrins belong to the family of cell adhesion molecules and are involved in tumor angiogenesis. Multiple subtypes of integrin are highly expressed in BTCs (86, 87). Integrin as a receptor can promote endothelial cell migration by activating FAK and MAPK signaling pathways. Moreover, integrin on the surface of vascular endothelial cells can bind to a certain structure of the extracellular matrix to accelerate the migration of endothelial cells and the formation of tumor blood vessels (88). Cilengitide is an integrin antagonist that can recognize and interact with integrins. Their interaction induces tumor cell apoptosis and inhibits tumor angiogenesis (89). Thalidomide can also inhibit angiogenesis by blocking the secretion of VEGF and basic fibroblast growth factor (bFGF) (90).

Vasculogenic mimicry (VM) is a form of vessels that is different from vessels derived from classic tumor angiogenesis and is independent of the vascular endothelium (91). It is composed of a cord formed by aggressive and poorly differentiated tumor cells, through which blood can be seen (92). The pipeline is connected with the host’s blood vessels so that the tumor cells can obtain blood supply to meet the needs of tumor growth, invasion and metastasis (93). VM has been found in liver cancer, lung cancer, prostate cancer and ovarian cancer, and it has been proven to correlate with tumor growth, differentiation and invasion. However, there is still a lack of research in BTCs.

## Clinical Progress of Antiangiogenic Therapy in BTCs

**Figure 3:**
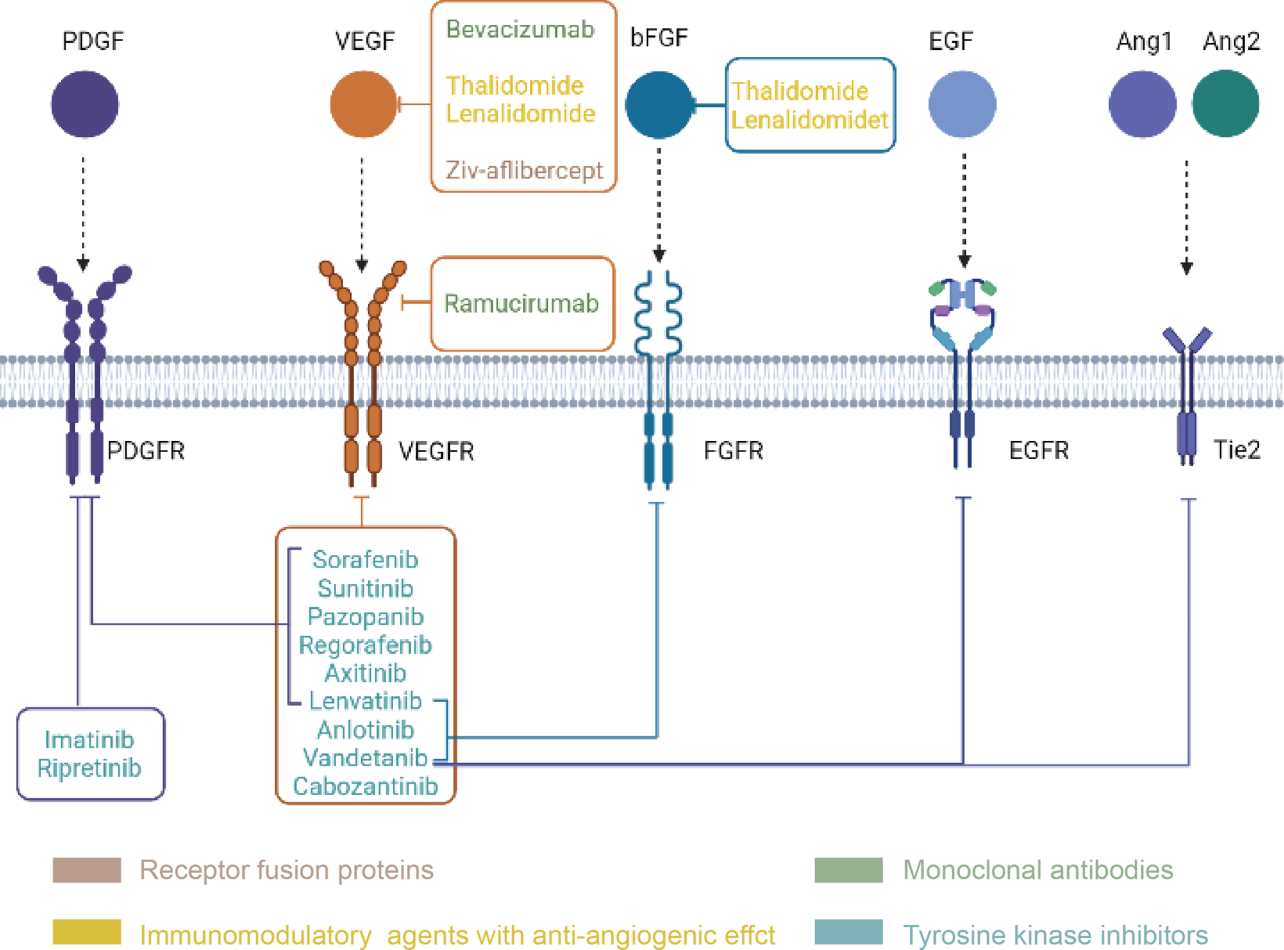
Classification and targets of anti-angiogenic drugs (Created in BioRender.com). Every drug in the picture points to its own targets, and the four drug types are distinguished by four colors.

Anti-angiogenic drugs (**Figure 3**) are currently divided into three categories: anti-VEGF monoclonal antibodies such as bevacizumab; signaling pathway inhibitors, represented by the small molecule tyrosine kinase inhibitors; and recombinant human vascular endostatin (94). Bevacizumab monotherapy has been shown to be less effective. Long-term clinical studies have shown that bevacizumab monotherapy can prolong progression-free survival (PFS) but not overall survival (OS), suggesting that inhibition of the classic VEGF pathway may activate compensatory pathways that promote tumor angiogenesis or metastasis, thereby leading to a rebound in tumor malignancy. Small molecule multitargeted receptor tyrosine kinase (RTK) inhibitors such as solafinib can simultaneously suppress multiple signaling pathways (95). This effect is expected to solve the problems of the abnormal activation of other signaling pathways in the case of a single inhibition of VEGF signaling pathways. Although TKI drugs, including sorafenib, axitinib, and sunitinib, are all multitarget inhibitors, their effects are mostly the same (96, 97). For example, the main targets of axitinib are VEGFR-1, VEGFR-2, VEGFR-3, the role of those are mainly angiogenesis (98). The inhibitory effect is not obvious on the target of whose roles are mainly promoting tumor cell survival and proliferation. Besides, these small molecule tyrosine kinase inhibitors have minimal effect on the tumor microenvironment. To improve the efficacy, on the one hand, a combination of drugs can be applied to interfere with angiogenesis and tumor proliferation simultaneously; on the other hand, a new generation of antiangiogenic drugs should cover more targets involved in the tumor growth. Anlotinib, as a new generation of antiangiogenic drugs, can inhibit tumor angiogenesis by targeting VEGFR, FGFR and PDGFR, meanwhile, it can also inhibits tumor growth by targeting c-kit (99). The significantly prolonged PFS and OS achieved in anlotinib treated drug-resistant NSCLC patients may be due to this dual effect (100). Currently, antiangiogenesis-based drug strategies for the treatment of malignant tumors include single-drug therapies with targeted drugs, combinations of chemotherapy and targeted drugs, combinations of immunotherapy and targeted drugs, and combined applications of targeted drugs. At present, antiangiogenic therapy has not been approved for the clinical treatment of biliary tract tumors, and most of the relevant studies are in the clinical trial stage. The following will summarize the research progress of antiangiogenic therapy for biliary tumors (**Table 1**).

**Table 1:** Anti-angiogenic drugs of BTCs in phase II.

Therapy	Target	NCT	Line	Phase	n	Patients	mPFS/mTTP months (95% CI)	mOS months ( 95% CI)	ORR	DCR
***monotherapies***										
Sorafenib	VEGFR, PDGFR, Raf	NCT 00238212	first	II	31	advanced BTC	3.0 (2.0-4.0)	9.0 (4.0-12.0)	0.0%	39.0%
Sunitinib	VEGFR, PDGFR, c-Kit, IRE1α	NCT 01718327	second	II	53	advanced iCCA	5.2	9.6 (5.8-13.1)	15.0%	85.0%
Sunitinib	VEGFR, PDGFR, c-Kit, IRE1α	NCT 01082809	second	II	56	advanced BTC	1.7 (1.0-2.4)*	4.8 (3.8-4.8)	8.9%	50.0%
Lenvatinib	VEGFR, FGFR, PDGFR, Kit, RET	NCT 02579616	second	II	17	advanced BTC			6.0%	82.0%
Lenvatinib	VEGFR, FGFR, PDGFR, Kit, RET	NCT 02579616	second	II	26	advanced BTC	3.2 (2.79-7.23)	7.4 (4.5-11.3)	11.5%	85.0%
Regorafenib	VEGFR, PDGFR, Kit, RET, Raf	NCT 02053376	second	II	43	advanced BTC	3.9 (3.2-6.2)	8.0 (3.3-18.6)	11.0%	56.0%
Apatinib	VEGFR-2	NCT 03521219	second	II	24	advanced iCCA	3.2 (2.7-5.1)	8.3 (3.8-12.9)	20.8%	62.5%
Apatinib	VEGFR-2	NCT 03251443	second	II	26	advanced iCCA	2.0 (0.7-3.3)	9. 0 (4.6-13.4)	11.5%	50.3%
Vandetanib	VEGFR, EGFR, PDGFR, Tie-2, FGFR	NCT 00753675	first	II	56	advanced BTC	3.4 (2.3-5)	7.4 (6.1 -11.7)	3.6%	25.0%
***Cytotoxic + targeted therapies***										
GEM+ Vandetanib	VEGFR, EGFR, PDGFR, Tie-2, FGFR	NCT 00753675	first	II	57		3.7 (2.9-6.2)	9.2 (6.9-11.6)	19.3%	29.8%
GEM+ placebo					52	advanced BTC	4.8 (1.9-7.3)	9.9 (8.2-16.9)	13.5%	20.0%
GC+ Sorafenib	VEGFR, PDGFR, Raf	NCT 00919061	first	II	39	advanced BTC	6.5 (3.5-8.3)	14.4 (11.6-19.2)		
GemCap+ Bevacizumab	VEGF	NCT 01007552	first	II	50	advanced BTC	8.1 (5.3-9.9)	10.2 (7.5-13.7)	24.0%	72.0%
GEMOX+ Bevacizumab	VEGF	NCT 00361231	first + second	II	35	advanced BTC	7.0 (5.3-10.3)	12.7 (7.3-18.1)	40.0%	69.0%
GEM+L-OHPL+ Cape+Panitumumab	EGFR	NCT 01206049	first	II	45	advanced BTC	6.1 (5.8-8.1)	9.5 (8.3-13.3)	46.0%	
GEM+L-OHPL+ Cape+Bevacizumab	VEGF				43		8.2 (5.3-10.6)	12.3 (8.8-13.3)	18.0%	
GEM+placebo	VEGFR, PDGFR, RAF, KIT, FLT-3	NCT 00661830	first	II	36	advanced BTC	4.9 (3.5-7.7)	11.2	10.0%	90.0%
GEM+Sorafenib					41		3.0 (1.8-7.2)	8.4	14.0%	86.0%
GC+Cediranib	VEGFR, PDGFR, c-kit	NCT 00939848	first	II	62	advanced BTC	8.0 (6.5-9.3)	14.1 (10.2-16.4)	44.0%	78.0%
GC+placebo					62		7.4 (5.7-8.5)	11.9 (9.2-14.3)	19.0%	65.0%
***Targeted + targeted therapies***										
Lenvatinib+ Pembrolizumab	VEGFR, FGFR, PDGFR, Kit, RET, PD-1	NCT 03895970	second	II	32	advanced BTC	4.9 (4.7-5.2)	11.0 (9.6-12.3)	25.0%	78.1%
Sorafenib +Erlotinib	VEGFR, PDGFR, RAF, KIT, FLT-3, EGFR	NCT 01093222	first	II	34	advanced BTC	2.0 (2.0-3.0)	6.0 (3.0-8.0)	6.0%	35.0%
Bevacizumab +Erlotinib	VEGF +EGFR	NCT 00356889	first	II	49	advanced BTC	4.4 (3.0-7.8)*	9.9 (7.2-13.6)	12.0%	63.0%

*These data represent mTTP.

GEM, Gemcitabine; GC, Gemcitabine + Cisplatin; GemCap, Gemcitabine + Capecitabine; GEMOX, Gemcitabine + Oxaliplatin; L-OHPL, Oxaliplatin; Cape, Capecitabine; NCT, National Clinical Trial; mPFS, median progression-free survival; mTTP, median time to progression; mOS, median overall survival; ORR, objective response rate; DCR, disease control rate.

## Monotherapy Regimens

The single-drug regimens of antiangiogenic therapy mostly use signaling pathway inhibitors, which can act on multiple targets simultaneously. Although more targets can increase the efficacy, the possibility of the corresponding side effects is also increased. Sunitinib, a multitarget tyrosine kinase inhibitor that targets PDGFR, VEGFR, KIT, FLT-3 and RET, can inhibit not only tumor proliferation but also angiogenesis at the same time. Sunitinib has been shown to be effective in the treatment of several solid tumors. In a study of advanced biliary tract tumors, Jun Ho Yi et al. believed that sunitinib monotherapy had a poor clinical effect. The PFS was only 1.7 months, and the incidence of grade 3–4 toxicities was high (46.4%) (101). Dreyer C et al. reported three cases of ICC patients with tumor progression after receiving first-line chemotherapy and treated them with sunitinib. Reductions in tumor size and density were observed in all three cases; one achieved partial remission, and two achieved stable disease (SD). They concluded that sunitinib was well tolerated and had manageable side effects. Based on these encouraging results, they initiated a phase II clinical study of sunitinib for second-line treatment in patients with advanced intrahepatic cholangiocarcinoma who had received chemotherapy (102). Fifty-three patients were included in this study, of whom 15% achieved partial remission, 71% achieved disease stability, the median PFS (mPFS) was 5.2 months and the median OS (mOS) was 9.6 months [95% CI: 5.8–13.1] (103). Thus, sunitinib monotherapy shows promising activity in advanced intrahepatic cholangiocarcinoma. Sorafenib, a hot multitarget tyrosine kinase inhibitor that targets VEGFR-2/-3, PDGFR-B, B-Raf, and C-Raf, achieved a 2% disease remission rate in the second-line treatment of 46 patients with advanced cholangiocarcinoma. The median progression-free survival was 2.3 months (range: 0–12 months), and the median overall survival was 4.4 months (range: 0–22 months) (104). Another study using sorafenib as a first-line regimen for advanced biliary tumors showed that the median PFS was 3 months (95% CI: 2–4 months) and the median OS was 9 months (95% CI: 4–12 months) (105). The results of these two clinical trials indicate that sorafenib did not achieve positive resultes in the treatment of advanced biliary tumors as a Monotherapy, and the combination therapy of sorafenib with other drugsit may be a promissing future direction. Lenvatinib is an inhibitor of VEGFR, FGFR and PDGFR. A phase II clinical trial was conducted with lenvatinib as a single agent for advanced biliary tract tumors. An interim evaluation of 17 patients showed a DCR of 82% (106). Finally, 26 patients were recruited into the study, and the results showed that the median PFS was 3.19 months (95% CI: 2.79–7.23) and the median OS was 7.35 months (95% CI: 4.50–11.27). Therefore, the study authors concluded that lenvatinib showed promising therapeutic effects in advanced biliary tract tumors with manageable side effects (107). Regorafenib is a multitarget inhibitor that targets VEGFR, PDGFR-β, KIT, RET and RAF-1. For patients with advanced biliary tract tumors who received failing first-line treatment, the average progression-free survival of regorafenib treatment was 3.9 months (95% CI: 3.2–6.2), and the average overall survival time was 8 months (95% CI: 3.3–18.6). Regorafenib may exert its promising efficacy in advanced biliary tract tumors, which is worthy of further study (108).

## Combination Therapy Regimens

### Chemotherapy Combined With Targeted Therapy

A phase II study of bevacizumab combined with gemcitabine/capecitabine in the treatment of advanced BTCs showed that the mPFS was 8.1 months, and the mOS was 10.2 months. Compared with the results of the gemcitabine/cisplatin combination in the ABC-02 trial (8 months of PFS and 11.7 months of OS), it could not be concluded that bevacizumab combined with Gemcitabine plus Capecitabine (GemCap) would benefit patients. However, more clinical stage IV patients were enrolled in the former group than in the latter group, and the proportion of patients with stage III disease was relatively low (6% vs. 25%) (109). Gemcitabine plus Oxaliplatin (GEMOX) in BTCs achieved an ORR of 26%-50% and a median overall survival of 11–12 months. The ORR of Gemox-B (the addition of bevacizumab to GEMOX) in BTCS reached 40%, the median progression-free survival reached 7.0 months, and the median overall survival reached 12.7 months (110). A phase II clinical trial showed that after two treatment cycles of bevacizumab combined with gemcitabine and oxaliplatin, the maximum standardized uptake value on FDG-PET scans was significantly decreased, indicating disease control and longer PFS and OS (110). A phase II clinical trial of the multitarget tyrosine kinase inhibitor cediranib combined with first-line chemotherapy in the treatment of advanced biliary tract cancer was conducted. Patients were randomly divided into two groups: cediranib combined with chemotherapy or placebo combined with chemotherapy. However, only ORR but not mPFS or mOSz showed a statistically significant difference, with 44% in the cediranib group versus 19% in the placebo group (111). In addition, sorafenib has also been introduced into several clinical trials of combination therapy regimens. However, the strategy of sorafenib combined with gemcitabine and cisplatin failed to improve the efficacy (112). Another trial on sorafenib combined with gemcitabine versus gemcitabine combined with placebo showed that the addition of sorafenib did not improve the outcome of advanced biliary tumors (113). Vandetanib is also a kinase inhibitor that has been shown to inhibit angiogenesis *in vivo* and *in vitro*. The efficacy and side effects of vandetanib as a monotherapy, vandetanib combined with gemcitabine and gemcitabine combined with placebo were studied. There was no difference in side effects among the three groups, while the objective response rate (ORR) of the vandetanib combined with gemcitabine group was higher than those of the other two groups (114). The efficacy of chemotherapy combined with EGFR inhibitor and VEGF monoclonal antibody was also studied, and the results showed no significant difference in mOS and mPFs between the GEM + L-OHPL + CAPE + panitumumab group and the GEM + L-OHPL + CAPE + bevacizumab group, although the former group had a higher ORR (115). From the results above, a conclusion can be drawn that cytotoxic drugs combined with anti-angiogenic drugs may achieve a better ORR in patients with BTCs, although little improvements in mPFS and mOS was observed.

### Combination Therapy With Multiple Targeted Drugs

A phase II multicenter clinical trial of bevacizumab and erlotinib (an EGFR tyrosine kinase inhibitor) showed that 12% of patients had a confirmed partial response, 51% achieved stable disease, the median TTP was 4.4 months (95% CI: 3.0–7.8), and the median overall survival was 9.9 months (95% CI: 7.2–13.6) (116). Another clinical trial recruited 32 patients who were pretreated with systemic antitumor treatments to receive treatment with pembrolizumab combined with lenvatinib. The ORR of all patients was 25%, the median OS was 11.0 months (95% CI: 9.6–12.3), and the median PFS was 4.9 months (95% CI: 4.7–5.2) (117). Furthermore, a phase II SWOG study with sorafenib and erlotinib for advanced cholangiocarcinoma yielded a median progression-free survival of 2 months (95% CI: 2–3 months) and a median overall survival of 6 months (95% CI: 3–8 months) (118). The combination of multiple targeted drugs as an alternative strategy for advanced BTCs has preclinical evidences. The activity of this combination has been verified in some clinical trails, Although, the improvement in OS or PFS should be investigated by further clinical trails.

## Discussion

Biliary tract tumors account for 3% of all digestive tract tumors. Their incidence has been increasing in recent years for various reasons (119). Therefore, it is urgent to increase the treatment selection of BTCs (120, 121). The development of individualized treatment plans is of great significance for cholangiocarcinoma with high heterogeneity (122, 123). The clinical trial results revealed that most antiangiogenic drugs alone or in combination did not significantly prolong OS and PFS in patients with biliary tract tumors. However, some of these clinical trials achieved considerable ORR, indicating significant tumor regression at the early stage of treatment. There are two possible reasons for the unsatisfactory results of antiangiogenic therapy. On the one hand, a compensatory feedback pathway may be activated when one angiogenesis pathway is inhibited. For example, inhibiting VEGF signaling pathways may activate Ang pathways. On the other hand, inhibition of angiogenesis leads to hypoxia in the local environment, which may promote the expression of tumor proliferation and migration genes. For example, HIF-1 and downstream gene expression levels were increased after VEGF inhibition. Therefore, combination therapy as the future direction of antiangiogenic therapy seems to be feasible. The combination of the antiangiogenic drug bevacizumab and the antiproliferative drug acetazolamide results in tumor inhibition in mice (124). Further clinical studies should be performed in the future to test this hypothesis. Based on the theory that antiangiogenic agents help target drugs penetrate into the tumor microenvironment, the effect of pemigatinib, a novel drug, which is approved by the FDA for the treatment of cholangiocarcinoma, in combined with should also be investigated for the treatment of patients with FGFR mutations.

The selection of potential patients who will benefit from antiangiogenic drugs is also crucial for the design of clinical trials and may be a critical factor for reaching the main endpoint. Dreyer C et al. reported promising results in three iCCA patients who were selected with hypervascular features. Therefore, the selection of cases with active tumor angiogenesis through imaging or histological evaluation may be conducive to improving the ORR. Some researchers have confirmed that the DWI phase of MRI is effective in evaluating tumor angiogenesis (125). Wu Xin et al. analyzed 88 cases of eCCA and found that ADC values were negatively correlated with MVD and VEGF (p < 0.05), indicating that DWI could be performed for the selection of BTC patients who may benefit from antiangiogenic treatment. It is critical to find more useful methods and markers to help clinicians assess whether patients can benefit from antiangiogenic therapy before medication.

At present, in clinical studies, RECIST is mostly applied to evaluate the effect of antiangiogenic therapy. However, antiangiogenic treatment may not result in a significant change in tumor volume in BTCs. After vascular degeneration, the interior of the tumor is necrotic, and the volume may not change. Therefore, traditional RECIST is not appropriate to evaluate the efficacy. Some researchers have proposed new ways to assess efficacy. It has been suggested that activated circulating vascular epithelial cells (aCECs) have higher sensitivity and reliability in efficacy evaluation than upstream factors such as VEGF. Some scholars have proposed using intratumor blood perfusion indicators [such as blood flow (BF) and blood volume (BV)] to reflect changes in blood supply. In particular, in view of the theory of vascular normalization proposed in recent years, how to evaluate the time window of anti-vascular therapy has become an urgent problem to be solved.

There are many kinds of cells in the tumor microenvironment, and the cytokines secreted and the receptors expressed by them constitute multiple signaling pathways that interact with each other. These signaling pathways are involved in tumor angiogenesis (126). Inhibition of one target or one pathway may result in short-term tumor regression, but inhibition of one pathway may promote another pathway. Therefore, effective antiangiogenic therapy needs to focus on common targets or multiple targets of multiple pathways and solve drug delivery problems in the leaky and poorly perfused tumor microenvironment. The study of improving the mechanism of angiogenesis in cholangiocarcinoma is helpful to find new therapeutic targets. Strengthening the construction of methods to evaluate tumor vascular normalization is helpful for the clinical development of reasonable antivascular therapy.

In conclusion, as highly heterogeneous tumors, the treatment and management of BTCs needs comprehensive evaluation and individualized medication, for which antiangiogenic therapy is a promising treatment method.

## Author Contributions

YW collected and analyzed the previous research fruits, and wrote the manuscript. TC, KL, and WM modified this article. ZL, AS, JL, WZ, SL, SH and CP contributed to the search for literature. ZZ designed and supervised the review. All authors contributed to the article and approved the submitted version.

## Funding

Our study was supported by Shandong University Multidisciplinary Research and Innovation Team of Young Scholars (Grant No. 2020QNQT002), National Natural Science Foundation of China (Grant No. 82072676, 82172791), China Postdoctoral Science Foundation(Grant No. 2020M682190, 2020M682195), Clinical Research Foundation of Shandong University(Grant No. 2020SDUCRCA018), Natural Science Foundation of Shandong Province(ZR2019MH008), Jinan City Science and Technology Development Program (Grant No. 201805017, 201805013), Clinical Research Innovation Fund Project (CXPJJH11800001-2018240), Hengrui Hepatobiliary and Pancreatic Foundation (Grant No.Y-2017-144), and Beijing Medical Award Foundation(YXJL-2020-0785-0967, YXJL-2020-0785-0968).

## Conflict of Interest

The authors declare that the research was conducted in the absence of any commercial or financial relationships that could be construed as a potential conflict of interest.

The reviewer ZL declared a shared affiliation, with no collaboration, with TC to the handling editor at the time of review.

## Publisher’s Note

All claims expressed in this article are solely those of the authors and do not necessarily represent those of their affiliated organizations, or those of the publisher, the editors and the reviewers. Any product that may be evaluated in this article, or claim that may be made by its manufacturer, is not guaranteed or endorsed by the publisher.

## Abbreviations

BTCs, Biliary tract cancers; CCA, Cholangiocarcinoma; iCCA, intrahepatic cholangiocarcinoma; pCCA, perihilar cholangiocarcinoma; dCCA, distal cholangiocarcinoma; eCCA, extrahepatic cholangiocarcinoma; GC, gallbladder cancer; DCR, disease control rate; OS, overall survival; PFS, progress free survival; mPFS, median progression-free survival; mTTP, median time to progression; mOS, median overall survival; ORR, objective response rate; MVD, microvessel density; ROS, reactive oxygen species; VEGF, Vascular Endothelial Growth Factor; VEGFR, Vascular Endothelial Growth Factor Receptor; RTK, tyrosine kinases; MMPs, matrix metalloproteinases; TGFβ1, Transforming Growth Factor Beta 1; TNF-α, Tumor necrosis factor-α; PDGF, The platelet-derived growth factor family; PDGFR-β, platelet-derived growth factor; Ang1, Angiogenin-1; Ang2, Angiogenin-2; bFGF, basic fibroblast growth factor; HPC, hepatic stem/progenitor cells; PBC, primary biliary cirrhosis; Tems, TIE-2-expend monocytes; VM, Vasculogenic mimicry; RTKs, receptor tyrosine kinase; aCECs, activated circulating vascular epithelial cells; RECIST, Response Evaluation Criteria in Solid Tumours; BF, bloodflow; BV, blood volume; GEM, Gemcitabine; GC, Gemcitabine + Cisplatin; GemCap, Gemcitabine + Capecitabine; GEMOX, Gemcitabine + Oxaliplatin; GEMOX-B, Gemcitabine + Oxaliplatin + Bevacizumab; L-OHPL, Oxaliplatin; Cape, Capecitabine.

## References

[1] Labib PL Goodchild G Pereira SP. Molecular Pathogenesis of Cholangiocarcinoma. BMC Cancer (2019) 19(1). doi: 10.1186/s12885-019-5391-0 PMC6394015 30819129

[2] Valle JW Kelley RK Nervi B Oh DY Zhu AX. Biliary Tract Cancer. Lancet (2021) 397(10272):428–44. doi: 10.1016/S0140-6736(21)00153-7 33516341

[3] Banales JM Cardinale V Carpino G Marzioni M Andersen JB Invernizzi P. Expert Consensus Document: Cholangiocarcinoma: Current Knowledge and Future Perspectives Consensus Statement From the European Network for the Study of Cholangiocarcinoma (ENS-CCA). Nat Rev Gastroenterol Hepatol (2016) 13(5):261–80. doi: 10.1038/nrgastro.2016.51 27095655

[4] Marin JJG Prete MG Lamarca A Tavolari S Landa-Magdalena A Brandi G. Current and Novel Therapeutic Opportunities for Systemic Therapy in Biliary Cancer. Br J Cancer (2020) 123(7):1047–59. doi: 10.1038/s41416-020-0987-3 PMC7525457 32694694

[5] Banales JM Marin JJG Lamarca A Rodrigues PM Khan SA Roberts LR. Cholangiocarcinoma 2020: The Next Horizon in Mechanisms and Management. Nat Rev Gastroenterol Hepatol (2020) 17(9):557–88. doi: 10.1038/s41575-020-0310-z PMC7447603 32606456

[6] Chen T Li K Liu Z Liu J Wang Y Sun R. WDR5 Facilitates EMT and Metastasis of CCA by Increasing HIF-1α Accumulation in Myc-Dependent and Independent Pathways. Mol Ther (2021) 29(6):2134–50. doi: 10.1016/j.ymthe.2021.02.017 PMC8178527 33601056

[7] Viallard C Larrivée B. Tumor Angiogenesis and Vascular Normalization: Alternative Therapeutic Targets. Angiogenesis (2017) 20(4):409–26. doi: 10.1007/s10456-017-9562-9 28660302

[8] Folkman J. Tumor Angiogenesis: Therapeutic Implications. N Engl J Med (1971) 285(21):1182–6. doi: 10.1111/1523-1747.ep12625746 4938153

[9] Chen Y Yu Y Ding G Ding H. Lymphangiogenic and Angiogentic Microvessel Density in Gallbladder Carcinoma. Hepatogastroenterology (2011) 58(105):20–5. doi: 10.1136/gut.2008.170811corr1 21510280

[10] Thelen A Scholz A Benckert C Schröder M Weichert W Wiedenmann B. Microvessel Density Correlates With Lymph Node Metastases and Prognosis in Hilar Cholangiocarcinoma. J Gastroenterol (2008) 43(12):959–66. doi: 10.1007/s00535-008-2255-9 19107340

[11] Thelen A Scholz A Weichert W Wiedenmann B Neuhaus P Gessner R. Tumor-Associated Angiogenesis and Lymphangiogenesis Correlate With Progression of Intrahepatic Cholangiocarcinoma. Am J Gastroenterol (2010) 105(5):1123–32. doi: 10.1038/ajg.2009.674 19997097

[12] Weinberg BA Xiu J Lindberg MR Shields AF Hwang JJ Poorman K. Molecular Profiling of Biliary Cancers Reveals Distinct Molecular Alterations and Potential Therapeutic Targets. J Gastrointest Oncol (2019) 10(4):652–62. doi: 10.21037/jgo.2018.08.18 PMC6657312 31392046

[13] Jain RK. Normalization of Tumor Vasculature: An Emerging Concept in Antiangiogenic Therapy. Science (2005) 307(5706):58–62. doi: 10.1126/science.1104819 15637262

[14] Murata R Nishimura Y Hiraoka M. An Antiangiogenic Agent (TNP-470) Inhibited Reoxygenation During Fractionated Radiotherapy of Murine Mammary Carcinoma. Int J Radiat Oncol Biol Phys (1997) 37(5):1107–13. doi: 10.1016/S0360-3016(96)00628-1 9169820

[15] Fenton BM Paoni SF Ding I. Effect of VEGF Receptor-2 Antibody on Vascular Function and Oxygenation in Spontaneous and Transplanted Tumors. Radiother Oncol (2004) 72(2):221–30. doi: 10.1016/j.radonc.2004.05.005 15297140

[16] Ma J Pulfer S Li S Chu J Reed K Gallo JM. Pharmacodynamic-Mediated Reduction of Temozolomide Tumor Concentrations by the Angiogenesis Inhibitor TNP-470. Cancer Res (2001) 61(14):5491–8. doi: 10.1016/S0165-4608(01)00481-2 11454697

[17] Lacerda Q Tantawi M Leeper DB Wheatley MA Eisenbrey JR. Emerging Applications of Ultrasound-Contrast Agents in Radiation Therapy. Ultrasound Med Biol (2021) 47(6):1465–74. doi: 10.1016/j.ultrasmedbio.2021.01.032 PMC8044052 33653626

[18] Padera TP Stoll BR Tooredman JB Capen D di Tomaso E Jain RK. Pathology: Cancer Cells Compress Intratumour Vessels. Nature (2004) 427(6976):695. doi: 10.1038/427695a 14973470

[19] Fong GH. Mechanisms of Adaptive Angiogenesis to Tissue Hypoxia. Angiogenesis (2008) 11(2):121–40. doi: 10.1007/s10456-008-9107-3 18327686

[20] Bottaro DP Liotta LA. Cancer: Out of Air Is Not Out of Action. Nature (2003) 423(6940):593–5. doi: 10.1038/423593a 12789320

[21] Gilkes DM Xiang L Lee SJ Chaturvedi P Hubbi ME Wirtz D. Hypoxia-Inducible Factors Mediate Coordinated RhoA-ROCK1 Expression and Signaling in Breast Cancer Cells. Proc Natl Acad Sci (2014) 111(3):E384–93. doi: 10.1073/pnas.1321510111 PMC3903228 24324133

[22] Thienpont B Steinbacher J Zhao H D'Anna F Kuchnio A Ploumakis A. Tumour Hypoxia Causes DNA Hypermethylation by Reducing TET Activity. Nature (2016) 537(7618):63–8. doi: 10.1038/nature19081 PMC5133388 27533040

[23] Chen Y Chang G Chen X Li Y Li H Cheng D. IL-6-miR-210 Suppresses Regulatory T Cell Function and Promotes Atrial Fibrosis by Targeting Foxp3. Mol Cells (2020) 43(5):438–47. doi: 10.14348/molcells.2019.2275 PMC7264473 32345003

[24] Kerber EL Padberg C Koll N Schuetzhold V Fandrey J Winningh S. The Importance of Hypoxia-Inducible Factors (HIF-1 and HIF-2) for the Pathophysiology of Inflammatory Bowel Disease. Int J Mol Sci (2020) 21(22):8551. doi: 10.3390/ijms21228551 PMC7697655 33202783

[25] Kumar V Gabrilovich DI. Hypoxia-Inducible Factors in Regulation of Immune Responses in Tumour Microenvironment. Immunology (2014) 143(4):512–9. doi: 10.1111/imm.12380 PMC4253499 25196648

[26] Lequeux A Noman MZ Xiao M Van Moer K Hasmim M Benoit A. Targeting HIF-1 Alpha Transcriptional Activity Drives Cytotoxic Immune Effector Cells Into Melanoma and Improves Combination Immunotherapy. Oncogene (2021) 40(28):4725–35. doi: 10.1038/s41388-021-01846-x PMC8282500 34155342

[27] Vasudev NS Reynolds AR. Anti-Angiogenic Therapy for Cancer: Current Progress, Unresolved Questions and Future Directions. Angiogenesis (2014) 17(3):471–94. doi: 10.1007/s10456-014-9420-y PMC4061466 24482243

[28] Seton-Rogers S. When Good Drugs do Bad Things. Nat Rev Cancer (2009) 9(4):228–9. doi: 10.1038/nrc2632

[29] Jain RK. Normalizing Tumor Vasculature With Anti-Angiogenic Therapy: A New Paradigm for Combination Therapy. Nat Med (2001) 7(9):987–9. doi: 10.1038/nm0901-987 11533692

[30] Chen P Bonaldo P. Role of Macrophage Polarization in Tumor Angiogenesis and Vessel Normalization: Implications for New Anticancer Therapies. Int Rev Cell Mol Biol (2013) 301:1–35. doi: 10.1016/B978-0-12-407704-1.00001-4 23317816

[31] Carmeliet P Jain RK. Principles and Mechanisms of Vessel Normalization for Cancer and Other Angiogenic Diseases. Nat Rev Drug Discov (2011) 10(6):417–27. doi: 10.1038/nrd3455 21629292

[32] Ebos JM Lee CR Cruz-Munoz W Bjarnason GA Christensen JG Kerbel RS. Accelerated Metastasis After Short-Term Treatment With a Potent Inhibitor of Tumor Angiogenesis. Cancer Cell (2009) 15(3):232–9. doi: 10.1016/j.ccr.2009.01.021 PMC4540346 19249681

[33] Kontos CD Willett CG. Inhibiting the Inhibitor: Targeting Vascular Endothelial Protein Tyrosine Phosphatase to Promote Tumor Vascular Maturation. J Natl Cancer Inst (2013) 105(16):1163–5. doi: 10.1093/jnci/djt199 23899554

[34] Rolny C Mazzone M Tugues S Laoui D Johansson I Coulon C. HRG Inhibits Tumor Growth and Metastasis by Inducing Macrophage Polarization and Vessel Normalization Through Downregulation of PlGF. Cancer Cell (2011) 19(1):31–44. doi: 10.1016/j.ccr.2010.11.009 21215706

[35] Sawada J Urakami T Li F Urakami A Zhu W Fukuda M. Small GTPase R-Ras Regulates Integrity and Functionality of Tumor Blood Vessels. Cancer Cell (2012) 22(2):235–49. doi: 10.1016/j.ccr.2012.06.013 PMC3422514 22897853

[36] Li W Quan YY Li Y Lu L Cui M. Monitoring of Tumor Vascular Normalization: The Key Points From Basic Research to Clinical Application. Cancer Manag Res (2018) 10:4163–72. doi: 10.2147/CMAR.S174712 PMC6175544 30323672

[37] Abdalla A Xiao L Ullah MW Yu M Ouyang C Yang G. Current Challenges of Cancer Anti-Angiogenic Therapy and the Promise of Nanotherapeutics. Theranostics (2018) 8(2):533–48. doi: 10.7150/thno.21674 PMC5743565 29290825

[38] Mukherjee A Madamsetty VS Paul MK Mukherjee S. Recent Advancements of Nanomedicine Towards Antiangiogenic Therapy in Cancer. Int J Mol Sci (2020) 21(2). doi: 10.3390/ijms21020455 PMC7013812 31936832

[39] Huang D Lan H Liu F Wang S Chen X Jin K. Anti-Angiogenesis or Pro-Angiogenesis for Cancer Treatment: Focus on Drug Distribution. Int J Clin Exp Med (2015) 8(6):8369–76. PMC4537982 26309490

[40] Dai J Rabie AB. VEGF: An Essential Mediator of Both Angiogenesis and Endochondral Ossification. J Dent Res (2007) 86(10):937–50. doi: 10.1177/154405910708601006 17890669

[41] Lange C Storkebaum E de Almodóvar CR Dewerchin M Carmeliet P. Vascular Endothelial Growth Factor: A Neurovascular Target in Neurological Diseases. Nat Rev Neurol (2016) 12(8):439–54. doi: 10.1038/nrneurol.2016.88 27364743

[42] Shibuya M. VEGF-VEGFR System as a Target for Suppressing Inflammation and Other Diseases. Endocr Metab Immune Disord Drug Targets (2015) 15(2):135–44. doi: 10.2174/1871530315666150316121956 25772179

[43] Ogasawara S Yano H Higaki K Takayama A Akiba J Shiota K. Expression of Angiogenic Factors, Basic Fibroblast Growth Factor and Vascular Endothelial Growth Factor, in Human Biliary Tract Carcinoma Cell Lines. Hepatol Res (2001) 20(1):97–113. doi: 10.1016/S1386-6346(00)00117-0 11282489

[44] Navaneethan U Gutierrez NG Jegadeesan R Venkatesh PG Poptic E Liu X. Vascular Endothelial Growth Factor Levels in Bile Distinguishes Pancreatic Cancer From Other Etiologies of Biliary Stricture: A Pilot Study. Dig Dis Sci (2013) 58(10):2986–92. doi: 10.1007/s10620-013-2764-0 23828141

[45] Tang D Nagano H Yamamoto H Wada H Nakamura M Kondo M. Angiogenesis in Cholangiocellular Carcinoma: Expression of Vascular Endothelial Growth Factor, Angiopoietin-1/2, Thrombospondin-1 and Clinicopathological Significance. Oncol Rep (2006) 15(3):525–32. doi: 10.3892/or.15.3.525 16465407

[46] Yoshikawal D Ojima H Iwasaki M Hiraoka N Kosuge TK. Clinicopathological and Prognostic Significance of EGFR, VEGF, and HER2 Expression in Cholangiocarcinoma. Br J Cancer (2008) 98(2):418–25. doi: 10.1038/sj.bjc.6604129 PMC2361442 18087285

[47] Sun XN Cao WG Wang X Wang Q Gu BX Yang QC. Prognostic Impact of Vascular Endothelial Growth Factor-A Expression in Resected Gallbladder Carcinoma. Tumour Biol (2011) 32(6):1183–90. doi: 10.1007/s13277-011-0221-2 21853312

[48] Mobius C Demuth C Aigner T Wiedmann M Wittekind C Mössner J. Evaluation of VEGF A Expression and Microvascular Density as Prognostic Factors in Extrahepatic Cholangiocarcinoma. Eur J Surg Oncol (2007) 33(8):1025–9. doi: 10.1016/j.ejso.2007.02.020 17400419

[49] Guedj N Zhan Q Perigny M Rautou PE Degos F. Comparative Protein Expression Profiles of Hilar and Peripheral Hepatic Cholangiocarcinomas. J Hepatol (2009) 51(1):93–101. doi: 10.1016/j.jhep.2009.03.017 19446907

[50] Benckert C Jonas S Cramer T Von Marschall Z Schäfer G Peters M. Transforming Growth Factor Beta 1 Stimulates Vascular Endothelial Growth Factor Gene Transcription in Human Cholangiocellular Carcinoma Cells. Cancer Res (2003) 63(5):1083–92. 12615726

[51] Pan S Hu Y Hu M Xu Y Chen M Du C. S100A8 Facilitates Cholangiocarcinoma Metastasis *via* Upregulation of VEGF Through TLR4/NF−κb Pathway Activation. Int J Oncol (2020) 56(1):101–12. doi: 10.3892/ijo.2020.4977 PMC6910197 31746424

[52] You Z Bei L Cheng LP Cheng NS. Expression of COX-2 and VEGF-C in Cholangiocarcinomas at Different Clinical and Pathological Stages. Genet Mol Res (2015) 14(2):6239–46. doi: 10.4238/2015.June.9.9 26125824

[53] Zhang J Han C Zhu H Song K Wu T. miR-101 Inhibits Cholangiocarcinoma Angiogenesis Through Targeting Vascular Endothelial Growth Factor (VEGF). Am J Pathol (2013) 182(5):1629–39. doi: 10.1016/j.ajpath.2013.01.045 PMC3644734 23608225

[54] Leng K Xu Y Kang P Qin W Cai H Wang H. Akirin2 Is Modulated by miR-490-3p and Facilitates Angiogenesis in Cholangiocarcinoma Through the IL-6/STAT3/VEGFA Signaling Pathway. Cell Death Dis (2019) 10(4):262. doi: 10.1038/s41419-019-1506-4 30886152 PMC6423123

[55] Cheng R Chen Y Zhou H Wang B Du Q Chen Y. B7-H3 Expression and its Correlation With Clinicopathologic Features, Angiogenesis, and Prognosis in Intrahepatic Cholangiocarcinoma. APMIS (2018) 126(5):396–402. doi: 10.1111/apm.12837 29696716

[56] Mancino A Mancino MG Glaser SS Alpini G Bolognese A Izzo L. Estrogens Stimulate the Proliferation of Human Cholangiocarcinoma by Inducing the Expression and Secretion of Vascular Endothelial Growth Factor. Dig Liver Dis (2009) 41(2):156–63. doi: 10.1016/j.dld.2008.02.015 PMC2626155 18395502

[57] Kangsamaksin T Chaithongyot S Wootthichairangsan C Hanchaina R Tangshewinsirikul C Svasti J. Lupeol and Stigmasterol Suppress Tumor Angiogenesis and Inhibit Cholangiocarcinoma Growth in Mice *via* Downregulation of Tumor Necrosis Factor-α. PloS One (2017) 12(12):e0189628. doi: 10.1371/journal.pone.0189628 29232409 PMC5726636

[58] Xu YF Liu ZL Pan C Yang XQ Ning SL Liu HD. HMGB1 Correlates With Angiogenesis and Poor Prognosis of Perihilar Cholangiocarcinoma *via* Elevating VEGFR2 of Vessel Endothelium. Oncogene (2019) 38(6):868–80. doi: 10.1038/s41388-018-0485-8 30177842

[59] Francis H DeMorrow S Venter J Onori P White M Gaudio E. Inhibition of Histidine Decarboxylase Ablates the Autocrine Tumorigenic Effects of Histamine in Human Cholangiocarcinoma. Gut (2012) 61(5):753–64. doi: 10.1136/gutjnl-2011-300007 PMC3244572 21873469

[60] Jaidee R Kongpetch S Senggunprai L Prawan A Kukongviriyapan U Kukongviriyapan V. Phenformin Inhibits Proliferation, Invasion, and Angiogenesis of Cholangiocarcinoma Cells *via* AMPK-mTOR and HIF-1A Pathways. Naunyn Schmiedebergs Arch Pharmacol (2020) 393(9):1681–90. doi: 10.1007/s00210-020-01885-3 32383028

[61] Franchitto A Onori P Renzi A Carpino G Mancinelli R Alvaro D. Expression of Vascular Endothelial Growth Factors and Their Receptors by Hepatic Progenitor Cells in Human Liver Diseases. Hepatobiliary Surg Nutr (2013) 2(2):68–77. doi: 10.3978/j.issn.2304-3881.2012.10.11 24570919 PMC3924658

[62] Manzat Saplacan RM Balacescu L Gherman C Chira RI Craiu A Mircea PA. The Role of PDGFs and PDGFRs in Colorectal Cancer. Mediators Inflamm (2017) 2017:4708076. doi: 10.1155/2017/4708076 28163397 PMC5259650

[63] Heldin CH Westermark B. Platelet-Derived Growth Factor: Three Isoforms and Two Receptor Types. Trends Genet (1989) 5(4):108–11. doi: 10.1016/0168-9525(89)90040-1 2543106

[64] Boonjaraspinyo S Boonmars T Wu Z Loilome W Sithithaworn P Nagano I. Platelet-Derived Growth Factor May be a Potential Diagnostic and Prognostic Marker for Cholangiocarcinoma. Tumor Biol (2012) 33(5):1785–802. doi: 10.1007/s13277-012-0438-8 22733151

[65] Pan S Hu Y Hu M Jian H Chen M Gan L. Platelet-Derived PDGF Promotes the Invasion and Metastasis of Cholangiocarcinoma by Upregulating MMP2/MMP9 Expression and Inducing EMT *via* the P38/MAPK Signalling Pathway. Am J Transl Res (2020) 12(7):3577–95. PMC7407735 32774720

[66] Duan HX Li BW Zhuang X Wang LT Cao Q Tan LH. TCF21 Inhibits Tumor-Associated Angiogenesis and Suppresses the Growth of Cholangiocarcinoma by Targeting PI3K/Akt and ERK Signaling. Am J Physiol Gastrointest Liver Physiol (2019) 316(6):G763–73. doi: 10.1152/ajpgi.00264.2018 30920845

[67] Augustin HG Koh GY Thurston G Alitalo K. Control of Vascular Morphogenesis and Homeostasis Through the Angiopoietin–Tie System. Nat Rev Mol Cell Biol (2009) 10(3):165–77. doi: 10.1038/nrm2639 19234476

[68] Suri C Jones PF Patan S Bartunkova S Maisonpierre PC Davis S. Requisite Role of Angiopoietin-1, a Ligand for the TIE2 Receptor, During Embryonic Angiogenesis. Cell (Cambridge) (1996) 87(7):1171–80. doi: 10.1016/S0092-8674(00)81813-9 8980224

[69] Maisonpierre PC Suri C Jones PF Bartunkova S Wiegand SJ Radziejewski C. Angiopoietin-2, a Natural Antagonist for Tie2 That Disrupts *In Vivo* Angiogenesis. Science (1997) 277(5322):55–60. doi: 10.1126/science.277.5322.55 9204896

[70] Atanasov G Hau HM Dietel C Benzing C Krenzien F Brandl A. Prognostic Significance of TIE2-Expressing Monocytes in Hilar Cholangiocarcinoma. J Surg Oncol (2016) 114(1):91–8. doi: 10.1002/jso.24249 27111031

[71] Voigtländer T David S Thamm K Schlué J Metzger J Manns MP. Angiopoietin-2 and Biliary Diseases: Elevated Serum, But Not Bile Levels Are Associated With Cholangiocarcinoma. PloS One (2014) 9(5):e97046. doi: 10.1371/journal.pone.0097046 24823366 PMC4019663

[72] Xu YF Yang XQ Lu XF Guo S Liu Y Iqbal M. Fibroblast Growth Factor Receptor 4 Promotes Progression and Correlates to Poor Prognosis in Cholangiocarcinoma. Biochem Biophys Res Commun (2014) 446(1):54–60. doi: 10.1016/j.bbrc.2014.02.050 24565842

[73] Su WC Shiesh SC Liu HS Chen CY Chow NH Lin XZ. Expression of Oncogene Products HER2/Neu and Ras and Fibrosis-Related Growth Factors bFGF, TGF-Beta, and PDGF in Bile From Biliary Malignancies and Inflammatory Disorders. Dig Dis Sci (2001) 46(7):1387–92. doi: 10.1023/A:1010619316436 11478488

[74] Eyries M Siegfried G Ciumas M Montagne K Agrapart M Lebrin F. Hypoxia-Induced Apelin Expression Regulates Endothelial Cell Proliferation and Regenerative Angiogenesis. Circ Res (2008) 103(4):432–40. doi: 10.1161/CIRCRESAHA.108.179333 18617693

[75] Kidoya H Ueno M Yamada Y Mochizuki N Nakata M Yano T. Spatial and Temporal Role of the Apelin/APJ System in the Caliber Size Regulation of Blood Vessels During Angiogenesis. EMBO J (2008) 27(3):522–34. doi: 10.1038/sj.emboj.7601982 PMC2241654 18200044

[76] Berta J Kenessey I Dobos J Tovari J Klepetko W Jan Ankersmit H. Apelin Expression in Human Non-Small Cell Lung Cancer: Role in Angiogenesis and Prognosis. J Thorac Oncol (2010) 5(8):1120–9. doi: 10.1097/JTO.0b013e3181e2c1ff 20581707

[77] Muto J Shirabe K Yoshizumi T Ikegami T Aishima S Ishigami K. The Apelin-APJ System Induces Tumor Arteriogenesis in Hepatocellular Carcinoma. Anticancer Res (2014) 34(10):5313–20. 25275024

[78] Hall C Ehrlich L Venter J O'Brien A White T Zhou T. Inhibition of the Apelin/Apelin Receptor Axis Decreases Cholangiocarcinoma Growth. Cancer Lett (2017) 386:179–88. doi: 10.1016/j.canlet.2016.11.025 PMC5510601 27894959

[79] Heo K Kim YH Sung HJ Li HY Yoo CW Kim JY. Hypoxia-Induced Up-Regulation of Apelin Is Associated With a Poor Prognosis in Oral Squamous Cell Carcinoma Patients. Oral Oncol (2012) 48(6):500–6. doi: 10.1016/j.oraloncology.2011.12.015 22285858

[80] Diakowska D Markocka-Mączka K Szelachowski P Grabowski K. Serum Levels of Resistin, Adiponectin, and Apelin in Gastroesophageal Cancer Patients. Dis Markers (2014) 2014:1–8. doi: 10.1155/2014/619649 PMC4094727 25049439

[81] Wan Y Zeng ZC Xi M Wan S Hua W Liu YL. Dysregulated microRNA-224/Apelin Axis Associated With Aggressive Progression and Poor Prognosis in Patients With Prostate Cancer. Hum Pathol (2015) 46(2):295–303. doi: 10.1016/j.humpath.2014.10.027 25532941

[82] Aishima S Taguchi K Sugimachi K Asayama Y Nishi H Shimada M. The Role of Thymidine Phosphorylase and Thrombospondin-1 in Angiogenesis and Progression of Intrahepatic Cholangiocarcinoma. Int J Surg Pathol (2002) 10(1):47–56. doi: 10.1177/106689690201000108 11927969

[83] Yuan R Li Y Yang B Jin Z Xu J Shao Z. LOXL1 Exerts Oncogenesis and Stimulates Angiogenesis Through the LOXL1-FBLN5/alphavbeta3 Integrin/FAK-MAPK Axis in ICC. Mol Ther Nucleic Acids (2021) 23:797–810. doi: 10.1016/j.omtn.2021.01.001 33614230 PMC7868718

[84] Cao Y Xue L. Angiostatin. Semin Thromb Hemost (2004) 30(1):83–93. doi: 10.1055/s-2004-822973 15034800

[85] Walia A Yang JF Huang YH Rosenblatt MI Chang JH Azar DT. Endostatin's Emerging Roles in Angiogenesis, Lymphangiogenesis, Disease, and Clinical Applications. Biochim Biophys Acta (2015) 1850(12):2422–38. doi: 10.1016/j.bbagen.2015.09.007 PMC4624607 26367079

[86] Phanthaphol N Somboonpatarakun C Suwanchiwasiri K Chieochansin T Sujjitjoon J Wongkham S. Chimeric Antigen Receptor T Cells Targeting Integrin αvβ6 Expressed on Cholangiocarcinoma Cells. Front Oncol (2021) 11. doi: 10.3389/fonc.2021.657868 PMC7982884 33763382

[87] Huang Y Kong Y Zhang L He T Zhou X Yan Y. High Expression of ITGA3 Promotes Proliferation and Cell Cycle Progression and Indicates Poor Prognosis in Intrahepatic Cholangiocarcinoma. BioMed Res Int (2018) 2018:1–9. doi: 10.1155/2018/2352139 PMC5817212 29511671

[88] Duro-Castano A Gallon E Decker C Vicent MJ. Modulating Angiogenesis With Integrin-Targeted Nanomedicines. Adv Drug Deliv Rev (2017) 119:101–19. doi: 10.1016/j.addr.2017.05.008 28502767

[89] Desgrosellier JS Cheresh DA. Integrins in Cancer: Biological Implications and Therapeutic Opportunities. Nat Rev Cancer (2010) 10(1):9–22. doi: 10.1038/nrc2748 20029421 PMC4383089

[90] D'Amato RJ Loughnan MS Flynn E Folkman J. Thalidomide Is an Inhibitor of Angiogenesis. Proc Natl Acad Sci USA (1994) 91(9):4082–5. doi: 10.1073/pnas.91.9.4082 PMC43727 7513432

[91] Zhang J Qiao L Liang N Xie J Luo H Deng G. Vasculogenic Mimicry and Tumor Metastasis. J Buon (2016) 21(3):533–41. 27569069

[92] Fernández-Cortés M Delgado-Bellido D Oliver FJ. Vasculogenic Mimicry: Become an Endothelial Cell “But Not So Much”. Front Oncol (2019) 9:803. doi: 10.3389/fonc.2019.00803 31508365 PMC6714586

[93] Wei X Chen Y Jiang X Peng M Liu Y Mo Y. Mechanisms of Vasculogenic Mimicry in Hypoxic Tumor Microenvironments. Mol Cancer (2021) 20(1):7. doi: 10.1186/s12943-020-01288-1 33397409 PMC7784348

[94] Mauriz JL González-Gallego J. Antiangiogenic Drugs: Current Knowledge and New Approaches to Cancer Therapy. J Pharm Sci (2008) 97(10):4129–54. doi: 10.1002/jps.21286 18200520

[95] Marisi G Cucchetti A Ulivi P Canale M Cabibbo G Solaini L. Ten Years of Sorafenib in Hepatocellular Carcinoma: Are There Any Predictive and/or Prognostic Markers? World J Gastroenterol (2018) 24(36):4152–63. doi: 10.3748/wjg.v24.i36.4152 PMC6158485 30271080

[96] Abdelgalil AA Alkahtani HM Al-Jenoobi FI. Sorafenib. Profiles Drug Subst Excip Relat Methodol (2019) 44:239–66. doi: 10.1016/bs.podrm.2018.11.003 31029219

[97] Grimaldi AM Guida T D'Attino R Perrotta E Otero M Masala A. Sunitinib: Bridging Present and Future Cancer Treatment. Ann Oncol (2007) 18 Suppl 6:vi31–4. doi: 10.1093/annonc/mdm221 17591828

[98] Kelly RJ Rixe O. Axitinib (AG-013736). Recent Results Cancer Res (2010) 184:33–44. doi: 10.1007/978-3-642-01222-8_3 20072829

[99] Shen G Zheng F Ren D Du F Dong Q Wang Z. Anlotinib: A Novel Multi-Targeting Tyrosine Kinase Inhibitor in Clinical Development. J Hematol Oncol (2018) 11(1):120. doi: 10.1186/s13045-018-0664-7 30231931 PMC6146601

[100] Han B Li K Wang Q Zhang L Shi J Wang Z. Effect of Anlotinib as a Third-Line or Further Treatment on Overall Survival of Patients With Advanced Non-Small Cell Lung Cancer: The ALTER 0303 Phase 3 Randomized Clinical Trial. JAMA Oncol (2018) 4(11):1569–75. doi: 10.1001/jamaoncol.2018.3039 PMC6248083 30098152

[101] Yi JH Thongprasert S Lee J Doval DC Park SH Park JO. A Phase II Study of Sunitinib as a Second-Line Treatment in Advanced Biliary Tract Carcinoma: A Multicentre, Multinational Study. Eur J Cancer (1990) (2011) 48(2):196–201. doi: 10.1200/jco.2011.29.15_suppl.e14653 22176869

[102] Dreyer C. Disease Control With Sunitinib in Advanced Intrahepatic Cholangiocarcinoma Resistant to Gemcitabine-Oxaliplatin Chemotherapy. World J Hepatol (2015) 7(6):910. doi: 10.4254/wjh.v7.i6.910 25937868 PMC4411533

[103] Neuzillet CSJFL. Sunitinib as Second-Line Treatment in Patients With Advanced Intrahepatic Cholangiocarcinoma (SUN-CK Phase II Trial): Safety, Efficacy, and Updated Translational Results. J Clin Oncol (2015) 33(3_suppl):343. doi: 10.1200/jco.2015.33.3_suppl.343

[104] Bengala C Bertolini F Malavasi N Boni C Aitini E Dealis C. Sorafenib in Patients With Advanced Biliary Tract Carcinoma: A Phase II Trial. Br J Cancer (2010) 102(1):68–72. doi: 10.1038/sj.bjc.6605458 19935794 PMC2813746

[105] El-Khoueiry AB Rankin CJ Ben-Josef E Lenz HJ Gold PJ Hamilton RD. SWOG 0514: A Phase II Study of Sorafenib in Patients With Unresectable or Metastatic Gallbladder Carcinoma and Cholangiocarcinoma. Invest New Drugs (2012) 30(4):1646–51. doi: 10.1007/s10637-011-9719-0 PMC3490705 21748296

[106] Morizane C Ueno M Sasaki T Nagashima F Mizuno N Shimizu S. Interim Analysis of a Phase 2 Study of Lenvatinib (LEN) Monotherapy as Second-Line Treatment in Unresectable Biliary Tract Cancer (BTC). Ann Oncol (2017) 35(4_suppl):310. doi: 10.1200/JCO.2017.35.4_suppl.310 PMC7667859 33198671

[107] Ueno M Ikeda M Sasaki T Nagashima F Mizuno N Shimizu S. Phase 2 Study of Lenvatinib Monotherapy as Second-Line Treatment in Unresectable Biliary Tract Cancer: Primary Analysis Results. BMC Cancer (2020) 20(1). doi: 10.1186/s12885-020-07365-4 PMC7667859 33198671

[108] Sun W Patel A Normolle D Patel K Ohr J Lee JJ. A Phase 2 Trial of Regorafenib as a Single Agent in Patients With Chemotherapy-Refractory, Advanced, and Metastatic Biliary Tract Adenocarcinoma. Cancer (2019) 125(6):902–9. doi: 10.1002/cncr.31872 PMC6402964 30561756

[109] Iyer RV Pokuri VK Groman A Ma WW Malhotra U Iancu DM. A Multicenter Phase II Study of Gemcitabine, Capecitabine, and Bevacizumab for Locally Advanced or Metastatic Biliary Tract Cancer. Am J Clin Oncol (2018) 41(7):649–55. doi: 10.1097/COC.0000000000000347 27849649

[110] Zhu AX Meyerhardt JA Blaszkowsky LS Kambadakone AR Muzikansky A Zheng H. Efficacy and Safety of Gemcitabine, Oxaliplatin, and Bevacizumab in Advanced Biliary-Tract Cancers and Correlation of Changes in 18-Fluorodeoxyglucose PET With Clinical Outcome: A Phase 2 Study. Lancet Oncol (2010) 11(1):48–54. doi: 10.1016/S1470-2045(09)70333-X 19932054

[111] Valle JW Wasan H Lopes A Backen AC Palmer DH Morris K. Cediranib or Placebo in Combination With Cisplatin and Gemcitabine Chemotherapy for Patients With Advanced Biliary Tract Cancer (ABC-03): A Randomised Phase 2 Trial. Lancet Oncol (2015) 16(8):967–78. doi: 10.1016/S1470-2045(15)00139-4 PMC4648082 26179201

[112] Lee JK Capanu M O'Reilly EM Ma J Chou JF Shia J. A Phase II Study of Gemcitabine and Cisplatin Plus Sorafenib in Patients With Advanced Biliary Adenocarcinomas. Br J Cancer (2013) 109(4):915–9. doi: 10.1038/bjc.2013.432 PMC3749586 23900219

[113] Moehler M Maderer A Schimanski C Kanzler S Denzer U Kolligs FT. Gemcitabine Plus Sorafenib Versus Gemcitabine Alone in Advanced Biliary Tract Cancer: A Double-Blind Placebo-Controlled Multicentre Phase II AIO Study With Biomarker and Serum Programme. Eur J Cancer (2014) 50(18):3125–35. doi: 10.1016/j.ejca.2014.09.013 25446376

[114] Santoro A Gebbia V Pressiani T Testa A Personeni N Arrivas Bajardi E. A Randomized, Multicenter, Phase II Study of Vandetanib Monotherapy Versus Vandetanib in Combination With Gemcitabine Versus Gemcitabine Plus Placebo in Subjects With Advanced Biliary Tract Cancer: The VanGogh Study. Ann Oncol (2015) 26(3):542–7. doi: 10.1093/annonc/mdu576 25538178

[115] Jensen LFEPJ. Randomized Phase II Crossover Trial Exploring the Clinical Benefit From Targeting EGFR or VEGF With Combination Chemotherapy in Patients With Non-Resectable Biliary Tract Cancer. J Clin Oncol (2015) 33(15_suppl):4071. doi: 10.1200/jco.2015.33.15_suppl.4071

[116] Lubner SJ Mahoney MR Kolesar JL Loconte NK Kim GP Pitot HC. Report of a Multicenter Phase II Trial Testing a Combination of Biweekly Bevacizumab and Daily Erlotinib in Patients With Unresectable Biliary Cancer: A Phase II Consortium Study. J Clin Oncol (2010) 28(21):3491–7. doi: 10.1200/JCO.2010.28.4075 PMC2917213 20530271

[117] Lin J Yang X Long J Zhao S Mao J Wang D. Pembrolizumab Combined With Lenvatinib as Non-First-Line Therapy in Patients With Refractory Biliary Tract Carcinoma. Hepatobiliary Surg Nutr (2020) 9(4):414–24. doi: 10.21037/hbsn-20-338 PMC7423565 32832493

[118] El-Khoueiry AB Rankin C Siegel AB Iqbal S Gong IY Micetich KC. S0941: A Phase 2 SWOG Study of Sorafenib and Erlotinib in Patients With Advanced Gallbladder Carcinoma or Cholangiocarcinoma. Br J Cancer (2014) 110(4):882–7. doi: 10.1038/bjc.2013.801 PMC3929880 24423918

[119] Oneda E Abu HM Zaniboni A. Biliary Tract Cancer: Current Medical Treatment Strategies. Cancers (Basel) (2020) 12(5). doi: 10.3390/cancers12051237 PMC7281170 32423017

[120] Qiu B Chen T Sun R Liu Z Zhang X Li Z. Sprouty4 Correlates With Favorable Prognosis in Perihilar Cholangiocarcinoma by Blocking the FGFR-ERK Signaling Pathway and Arresting the Cell Cycle. EBioMedicine (2019) 50:166–77. doi: 10.1016/j.ebiom.2019.11.021 PMC6921364 31761616

[121] Li Z Liu J Chen T Sun R Liu Z Qiu B. HMGA1-TRIP13 Axis Promotes Stemness and Epithelial Mesenchymal Transition of Perihilar Cholangiocarcinoma in a Positive Feedback Loop Dependent on C-Myc. J Exp Clin Cancer Res (2021) 40(1):86. doi: 10.1186/s13046-021-01890-1 33648560 PMC7923631

[122] Liu Z Sun R Zhang X Qiu B Chen T Li Z. Transcription Factor 7 Promotes the Progression of Perihilar Cholangiocarcinoma by Inducing the Transcription of C-Myc and FOS-Like Antigen 1. EBioMedicine (2019) 45:181–91. doi: 10.1016/j.ebiom.2019.06.023 PMC6642257 31248836

[123] Sun R Liu Z Qiu B Chen T Li Z Zhang X. Annexin10 Promotes Extrahepatic Cholangiocarcinoma Metastasis by Facilitating EMT *via* PLA2G4A/PGE2/STAT3 Pathway. EBioMedicine (2019) 47:142–55. doi: 10.1016/j.ebiom.2019.08.062 PMC6796529 31492557

[124] Vaeteewoottacharn K Kariya R Dana P Fujikawa S Matsuda K Ohkuma K. Inhibition of Carbonic Anhydrase Potentiates Bevacizumab Treatment in Cholangiocarcinoma. Tumor Biol (2016) 37(7):9023–35. doi: 10.1007/s13277-016-4785-8 26762407

[125] Meyer H Wienke A Surov A. Association Between VEGF Expression and Diffusion Weighted Imaging in Several Tumors—A Systematic Review and Meta-Analysis. Diagnostics (2019) 9(4):126. doi: 10.3390/diagnostics9040126 PMC6963772 31547581

[126] Weis SM Cheresh DA. Tumor Angiogenesis: Molecular Pathways and Therapeutic Targets. Nat Med (2011) 17(11):1359–70. doi: 10.1038/nm.2537 22064426

